# The feasibility of using arterial spin labeling for visualization of non perfused volumes after HIFU treatment in the kidney

**DOI:** 10.1186/2050-5736-3-S1-P60

**Published:** 2015-06-30

**Authors:** Frank Eschbach, Martijn de Greef, Joost Wijlemans, Chrit Moonen, Mario Ries

**Affiliations:** 1 University Medical Center Utrecht, Utrecht, Netherlands

## Background/introduction

MR guidance of High Intensity Focused Ultrasound (MFgHIFU) ablation offers the possibility to determine if complete tumor ablation has been established based on imaging the non-perfused volume (NPV). For thermal therapy, contrast enhanced (CE) imaging with a gadolinium-based contrast agent is currently the golden standard to quantify the NPV. A drawback of this method is that gadolinium based intra-vascular contrast agents interfere with HIFU therapy in two ways. First, ablation can release toxic free gadolinium, by long-term decomposition of the encapsulating chelate. Second, it degrades the quality of MR-thermometry. Therefore, even if residual viable tumor tissue is observed with CE-MRI, the treatment cannot be continued. To address this problem for HIFU interventions on the kidney, this study explores other means of perfusion imaging, such as Arterial Spin Labeling (ASL). Petros Martirosan’s group at the University of Tubingen used Flow-sensitive Alternating Inversion Recovery (FAIR) to image perfusion in the kidneys without the use of contrast agents. This *in vivo* study on a porcine model investigates if ASL potentially allows to perform NPV measurements during therapy and to continue the intervention if incomplete ablation of the target volume is observed.

## Methods

Image acquisition: An ablation in the renal cortex was performed in an *in vivo* porcine model. The animal was positioned decubitus right on a Sonalleve HIFU platform (Philips Healthcare, Vantaa, Finland), was under general anesthesia and mechanically ventilated. All scans were performed on a clinical 1.5T MR scanner (Achieva, Philips Healthcare, Best, The Netherlands). 7 treatment cells of 4 mm in diameter each were ablated in a honeycomb-like pattern. To image the NPV without the use of contrast agents, single shot gradient recalled echo-planar imaging of a coronal slice centered at the ablated region was preceded by slice selective inversion and global inversion (in an alternating fashion).

Imaging parameters: in-plane resolution: 3x3 mm2, slice thickness: 5mm, TE/TR: 44/4500 ms, inversion time: 1200 ms, matrix size: 128 x 128, number of dynamics: 20, slice selective inversion slice thickness: 10mm, non-selective inversion slice thickness: 300 mm. For the CE scan, a 3D FFE THRIVE sequence was used with an isotropic resolution of 1.5mm, a TR of 5.4 ms, a TE of 2.6 ms, a flip angle of 10, 2 averages and a reconstruction matrix of 512x512. Data analysis: Matlab (R2013b, The MathWorks, Inc., Natick, MA, USA) was used to realign the individual images, after which a subtraction per dynamic was performed. The subtractions were subsequently summed. Mevislab 2.4 (MeVis Medical Solutions AG, Bremen, Germany) was used to draw contours of NPV.

## Results and conclusions

**Figure 1:**
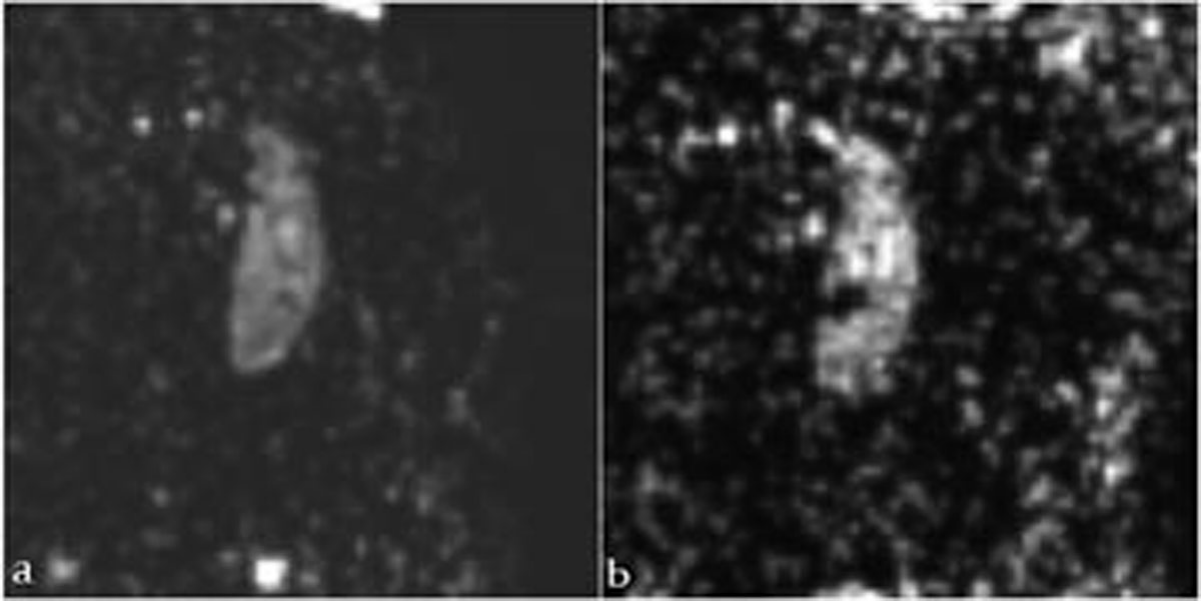
FAIR EPI image of the kidney, coronal orientation, before ablation (a) and after ablation (b). After ablation, the NPV is seen as a black volume on the left side of the kidney.

**Figure 2:**
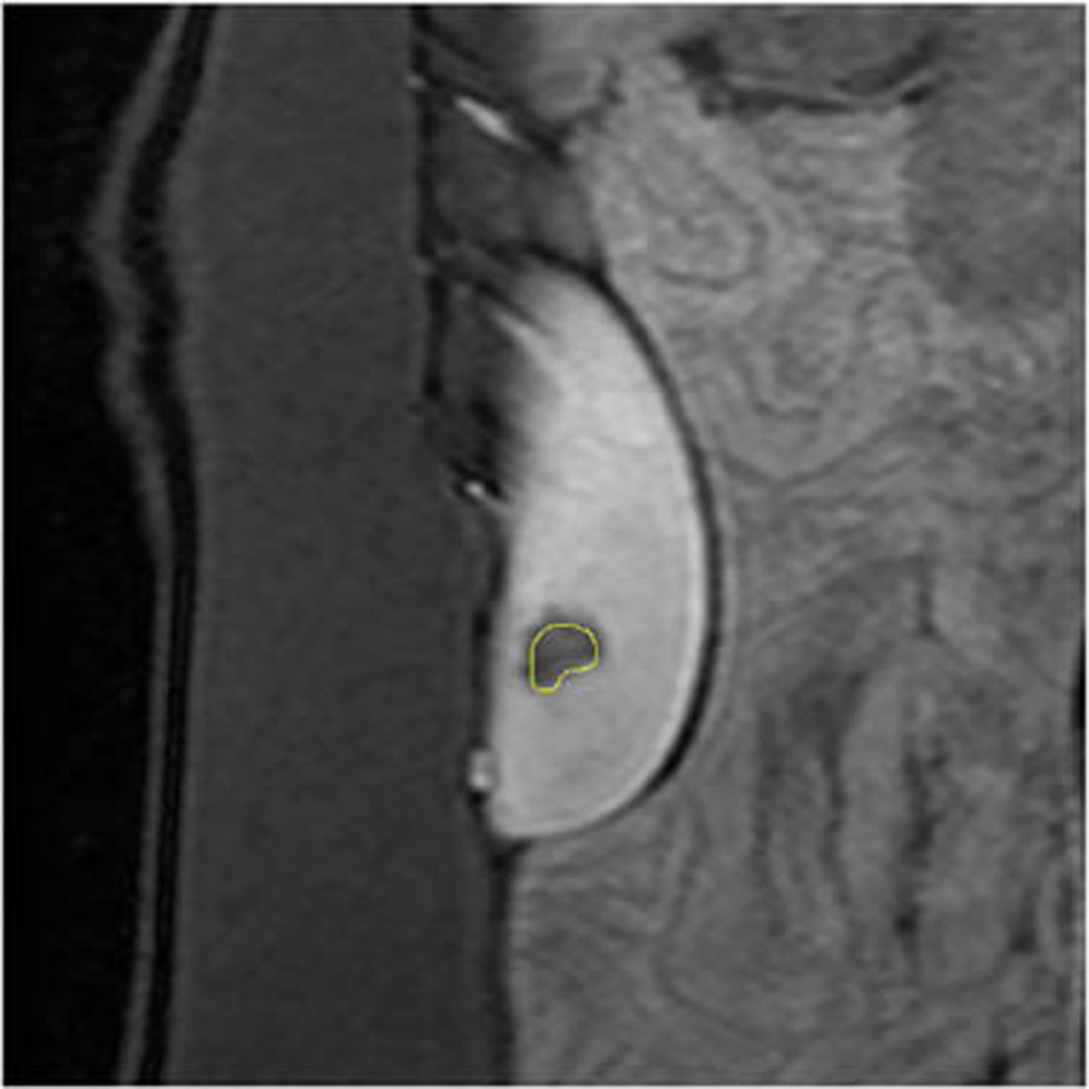
CE image, coronal orientation, The NPV is clearly visible as a black volume. The contour of the FAIR NPV is shown in yellow. Both the location and shape are comparable, unlike the size, which is smaller.

Figure 1 shows the resulting FAIR images before and after ablation. After ablation, the NVP is visible as a dark area at the left of the kidney. Figure 2 shows the corresponding CE image (golden standard). The yellow contour delineates the NPV as seen in the FAIR image. There is a clear correspondence in terms of location and shape in the NPVs as observed in both images. The NPV in the FAIR image is smaller than to the NPV in the CE image, which is, considering the difference in resolution, most likely due to a partial volume effect. The preliminary results show good correspondence between NPVs in the FAIR and CE images and therefore indicate FAIR to be a promising method to monitor the NPV progression in between the sonications. Future work is focused on an improved spatial resolution and a reduction of the required acquisition of the FAIR scans.

